# A Review of Depth of Focus in Measurement of the Amplitude of Accommodation

**DOI:** 10.3390/vision2030037

**Published:** 2018-09-06

**Authors:** David H. Burns, Peter M. Allen, David F. Edgar, Bruce J. W. Evans

**Affiliations:** 1 Institute of Optometry, 56-62 Newington Causeway, London SE1 6DS, UK; 2 School of Health and Social Care, London South Bank University, 103 Borough Road, London SE1 0AA, UK; 3 Department of Vision and Hearing Sciences & Vision and Eye Research Unit, Anglia Ruskin University, East Road, Cambridge CB1 1PT, UK; 4 Centre for Applied Vision Research, Division of Optometry and Visual Science, City, University of London, Northampton Square, London EC1V 0HB, UK

**Keywords:** amplitude of accommodation, depth of focus, measurement error, optometry

## Abstract

The aim of this review is to investigate the role of depth of focus (DoF) as a potential confounding variable in the measurement of the amplitude of accommodation (AoA). The role of DoF in human vision is briefly summarised, and it is noted that the prevalent method of measuring AoA is the push-up method. Factors influencing the effect of DoF on the push-up and other methods of measuring AoA are reviewed in detail. DoF is shown to add substantial measurement error in the routine assessment of accommodation when the AoA is measured by methods involving subjective judgement of an object’s clarity. Reliable compensation for this source of error is not realistically possible because of the complexity of the aetiology of DoF, and its inter-individual and intra-individual variation. The method of measurement also influences the extent of the error. It is concluded that methods of measurement of AoA that exclude DoF should be preferred.

## 1. Introduction

The principal purpose of clinical refraction is to reduce problems arising from refractive error. To do this, optometrists routinely question patients, repeatedly, about awareness of refractive blur. Therefore, it is important for the clinician to understand the depth of focus (DoF) of the human visual system, which is defined by Charman [1] as the range of an object’s vergence at the eye without any blur being detected.

**Figure 1:**
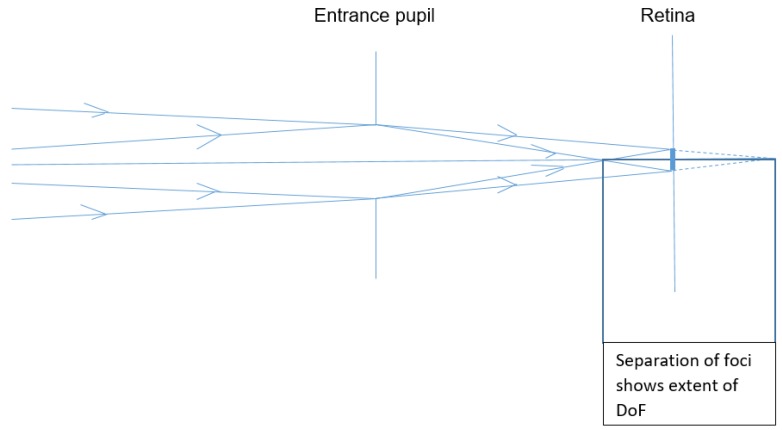
Schematic diagram illustrating the depth of focus (DoF) of the eye.

Figure 1 shows a schematic eye refracting two rays 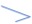 and 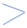 of differing vergences, which both form the same blur circle of diameter 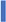 on the retina. If the blur circle is small enough for the observer to ignore, the depth of focus will be the difference between the two vergences at the entrance pupil.

Several factors together cause DoF in any optical focussing system. These factors include the following:

- Inherent imprecision in image formation, due to diffraction and aberration as explained by Lipson et al. [2]
- Non-inherent imprecision of focussing, due to suboptimal production of the physical elements of the focussing system
- Limitations to the detection of blur, as discussed by Wang and Ciuffreda [3].

For human vision, neural and perceptual factors combine with those given above to cause DoF. These factors include chronic uncorrected refractive error, which may reduce awareness of blur—an effect termed “neurological and perceptual tolerance”, that is discussed by Wang and Ciuffreda [3].

DoF has been found to vary extensively between individuals [4], and with viewing conditions, such as test-object luminance [5], and pupil diameter [6]. It may also be influenced by refractive history as this may lead to adaptation to blur [7].

Therefore, DoF in human vision is the result of a wide range of factors, some of which can be measured or estimated more reliably than others. Furthermore, some, such as diffraction, cannot be eliminated, and others, such as pupil diameter, vary. Hence DoF varies, and cannot be accurately estimated.

Optometrists in the UK measure the range of refractive power of the eye [8,9]. This range is termed the eye’s amplitude of accommodation (AoA). It is also measured by clinicians in other eye-care professions.

AoA does not include DoF. However, most current measurement of AoA is by methods that have the subjective expression of blur as the end-point but do not exclude DoF which therefore would cause the result to differ from the true value of AoA.

DoF may affect the measurement of AoA by any method that requires the recognition of blur. These methods, such as the push-up method, are widely used. Push-up was the first method for the measurement of AoA to be described, over 150 years ago, by Donders [10]. In clinical practice and in research, it has remained the prevalent method [11]. In the current review, the push-up method includes its variants, such as push-down.

## 2. Factors Influencing the Effect of DoF on the Measurement of AoA

In measuring AoA with any method affected by DoF, the measurement-error due to DoF is affected by factors including test-object parameters, pupil size, end-point criterion and perceptual discrimination. These factors are described in detail below.

Test-object parameters that influence DoF include the apparent size and the illuminance of the test object. DoF increases if test object detail subtends a larger angle at the eye [12], and decreases if the test object’s apparent brightness is decreased from comfortable levels [5]. Other parameters of the test object such as its colour [4], and its contrast [5], have also been found to influence DoF—although to a lesser extent. Factors that influence DoF have been reviewed and summarised [3].

In reviewing surveys of AoA, Kragha [13] noted that reporting of these parameters varied greatly. Most reports of AoA measurement specified test-object height only. The survey by Turner [14] specified the test object’s font and estimated luminance, and the study by Atchison et al. [15] reported measured luminance, contrast and colour. However, some cited references for AoA measurement specified no test-object parameters.

This lack of specification leads to difficulty in aggregating the results of different surveys of AoA. The relative validity of a survey’s results is reduced, to an unknown extent, by failure to report the level of a factor known to influence measurements. For example, when test-object illuminance is not specified, if two surveys of AoA were conducted identically except that in one the test object was illuminated less than in the other, the latter survey would be more reliable than the former.

Pupil size affects DoF considerably, as shown geometrically by Schwartz [16] in illustrating the concept of DoF, and empirically by Campbell [5]. The latter author discussed the differences between his results, and those predicted by geometry. However, pupil diameter cannot be easily controlled in clinical work, and changes rapidly under the influence of various factors. For example, the eye’s pupil diameter decreases, allowing greater DoF, with a variety of commonplace factors including increasing age [17], accommodation itself [18], mental effort [19], other cerebral factors [20], as well as any interactions of all these influences. Prediction, with adequate accuracy, of the overall effect of all these factors on DoF would be a particularly complex task, for which no literature reports were found.

Measurement of DoF would depend on the observer’s ability to perceive blur. The discrimination of minimal blur from sharpness would be the product of many factors, such as learning, adaptation, motivation, and eye health. The effects of those factors on blur discrimination, and the aggregation of those effects, are beyond the scope of this review. Furthermore, the end-point criterion for blur discrimination in AoA measurement has been specified differently by different authorities. It has ranged from minimum detectable blur [21,22], to resolution [23,24]. The criterion of minimum detectable blur is preferable as it is affected less by DoF. According to Wagstaff [25] it was the prevalent criterion in classic research into DoF.

Thus, DoF in human vision is multifactorial. This makes its influence on the measurement of AoA unpredictable.

## 3. Research Showing that DoF Affects Measurement of AoA

This section shows that AoA results were generally lower when measured using methods designed to reduce DoF than those found by research that did not appear to have set out to reduce DoF. The first such report was by Hamasaki et al. [26]. It compared measurements of AoA, using the push-up method, to those made by stigmatoscopy (a technique that uses the perceived sharpness of a spot of light to determine the refractive state of the eye and theoretically reduces DoF [27]). In this research, for each of the 106 participants, AoA measurements by stigmatoscopy were lower than those obtained by push-up, and the mean of all stigmatoscopy measurements was less than half of the mean push-up measurement. This was attributed to DoF contaminating the push-up readings. However, this research measured participants aged 41 to 60, so they would have had little or no accommodation [28], and therefore their findings may not be replicated in those outside the age range studied. Nonetheless, their findings were closely replicated in a study by Sun et al. [29], reporting a similar method, although with only seven participants but from age 12 to 46, with a mean age of 29 years.

The DoF of ten trained observers was measured in a study by Wagstaff [30] although the investigator noted that accuracy would be low because the causes of DoF were complex and varying. The investigator also measured the same participants’ AoA by the push-up method and by methods that reduced DoF (retinoscopy, and stigmatoscopy) observing that AoA measurements were higher with push-up by approximately the values found for DoF.

In a study by Atchison et al. [15], who looked at the effect of DoF on the measurement of accommodative amplitude by method-comparison, AoA was measured with the commonplace push-up method using test objects of constant real height. They were then made with smaller test objects, of constant apparent size, to reduce the measurements’ possible contamination by DoF. The former test objects were N5-size print and the latter were N3-size print if held at 40 cm, with the size adjusted in proportion to the viewing distance when positioned at other viewing distances. If the letters of constant apparent size had been upper-case they would have been 25% larger than median threshold resolution at 40 cm for their younger participants but they were lower-case so the size was close to the threshold of resolution, allowing less DoF than the N5 print. The 60 participants’ ages were 27 to 45 so the study did not address youthful accommodation. Results demonstrated that AoA measurements with reduced DoF were around 75% of those with N5 test objects; and around 55% of the most-cited [11] normative values of AoA [31] (those of Duane, in which he argued that “distinctness of near vision is not secured by a contraction of the pupil”).

Sergienko and Nikonenko [32] performed a similar study to that described above by Atchison et al. but with younger participants and without making measurements with a standard push-up method. Their 155 participants were aged 8 to 25. AoA was measured by the push-up method using test-objects of constant apparent size (58% of the maximum height of the text used by Atchison et al. [15] but these were four-way single Landolt Rings, so relatively legible). The values of AoA obtained were a similar proportion lower than established normative values as for Atchison’s study [15] with older participants described above.

This finding may be tempered by methodological differences. Duane’s participant group was far larger (4000 participants), and he used a fine line test-object whereas the other studies used text. He studied a much wider range of participant age; and used a moving target, whereas in the other studies, measurements were made while the target was stationary, thereby eliminating additional error due to reaction time (another source of measurement error, as described by Benzoni and Rosenfield [33]). Moreover, in Duane’s study but not in the studies with reduced DoF, homatropine cycloplegia (dosage and concentration not given) was used to measure refractive error of an unknown proportion of participants. Cycloplegia is a temporary reduction or paralysis of accommodation. and the effectiveness of homatropine for this purpose has been shown by Wolf and Hodge [34]. The AoA is the maximum increase in the eye’s power, from fully relaxed to maximum accommodation, so its clinical measurement involves relaxing accommodation as much as possible. Homatropine cycloplegia simplifies that by eliminating most or all accommodation. However, the difference in refraction between an individual’s lowest normal level of accommodation and the level under homatropine cycloplegia is not precisely predictable (and may be substantial), as demonstrated by Nayak et al. [35] showing that for young, normal eyes without high ametropia, homatropine 2% tended to relax accommodation beyond the lowest normal level by 0.33 D on average.

Studies that set out to measure AoA with reduced DoF may not have eliminated DoF, depending on the measurement technique used. Therefore, the disparity may be an underestimate. It may also be an underestimate because experimental conditions and participants’ expectations (participants included some experienced observers) would have differed from those of naive patients in routine clinical practice in the study by Duane [31]. For example, participants who were experienced observers would have been more likely than patients in routine clinical practice to recognise and express small degrees of blur.

Notwithstanding those methodological differences, the disparity is supported by other surveys of AoA. Established normative AoA values [11] were, on average, nearly double, compared to those obtained with reduced effects of DoF, as described above.

Other work has also supported the contention that DoF causes substantial error, sometimes tacitly, such as when AoA measurements were obtained by Woerhle et al. [36] for 25 participants, aged 10 to 40 years. The results were similar with the push-up and push-down methods, and the report cited other studies that found the same effect. The investigators did not consider the possible explanation, suggested by other authors [33,37], that the error due to DoF in push-down to recognition would have been substantially counterbalanced by the error due to reaction time in push-up.

Table 1 shows that DoF inflates measurement of AoA. Studies were excluded unless of relatively robust methodology, and involved at least twenty participants. The variability of the inflation is attributable to differences in the methods of reducing the DoF, since different methods may have varying degrees of residual DoF and other, differing, inherent sources of error.

In Table 1, the first two methods mentioned of reducing the DoF (reduced test-object subtense, and stigmatoscopy) are described above. The minus lens method requires the participant to resolve a test object viewed, through negatively powered lenses, which stimulate accommodation. It reduces DoF because the image of the test object, in the negative lens, generally subtends a smaller angle at the eye than is subtended by the test object in the commonplace push-up method. The other method referred to in Table 1, retinoscopy, is a more objective method of measuring the eye’s refractive power.

Five different methods of measuring AoA were compared on 31 participants, aged 31 to 53 years, in a study by Ostrin and Glasser [40]. The results showed general large variation between methods, between participants, and within participants. The investigators attributed some of the variation to the different influences of DoF on the different methods. They obtained the highest results with the push-up method, which, of the five methods compared, may have been the method most susceptible to influence by DoF. However, the report did not present data that would allow comparison of the degree to which the five methods were relatively prone to error from DoF.

## 4. Effect of the Method of Measurement

It has been shown empirically [12] that using optotypes of larger apparent size increases the variability of the results. The investigators found that this imprecision was attributable to DoF, and accordingly suggested that methods of measuring AoA be revised. However, most measurements of AoA in research reports such as those by Sterner et al. [41] and Adler et al. [42], and in clinical work, have been made by participants viewing text optotypes that vary in apparent size, sometimes as much as tenfold.

In the push-up method that is commonly used, measuring higher levels of AoA includes disproportionately higher errors. This is because, at higher levels of AoA, the test-object appears larger to the patient and is nearer, and the degree of defocus just sufficient to render an object indiscernible is directly proportional to the angular size of the object and inversely proportional to the object’s distance from the observer [12,43,44].

To overcome this increase of error with increased level of measurement, measurement with a test object of constant apparent size at different vergences was proposed and assessed [45]. This was achieved through using a Badal optometer system, as is in the study by Ostrin and Glasser [40] in comparing research methods of measuring AoA. However, investigations with differing methodologies [46,47] have shown that accommodation is significantly different if stimulated when viewing through a Badal system to when viewing in free space. Only one device using the Badal system in clinical measurement of AoA has been found. It was introduced in 1954 [48], and only mentioned [21] minimally in peer-reviewed literature. The device’s test-characters were so large that little accommodation would be required for any measurement, and its production was not sustained.

DoF causes the push-up method to give higher results for AoA than the minus-lens method [39,40,49,50]. DoF also affects measurements with the minus lens technique but less than in the push-up method [51], because a stationary object viewed through negatively-powered lenses appears smaller when that power increases.

The studies cited above all mentioned DoF as a possible cause of their higher AoA results with the push-up method, than with the minus-lens method, explaining that minus lenses reduce the apparent size of the test object more in measuring higher accommodation, so that DoF would have less effect on higher results. However, simple spectacle-magnification calculation shows that the effect would be far too small to account for the disparity in results between the two methods. Other subject characteristics such as age or refractive error may be involved. For example, it was found that school-age myopes accommodated less than age-matched non-myopes through minus-powered lenses [52].

If accommodation is measured using any method that requires the individual to recognise blur, the end-point could be anywhere between the degree of defocus that causes minimal blur and a degree that renders the test object indiscernible. For example, in the investigation by Atchison et al. [15] described above, participants were firmly instructed to say when first blur was seen, yet a review of their results suggests that their participants tended to interpret “first blur” as when the N5 print was blurred sufficiently to have become almost unrecognisable.

Furthermore, DoF accounted for accommodative response lagging almost a dioptre behind various levels of accommodative demand [53]. However, this investigation used an undefined end-point criterion of “objectionable blur”.

## 5. Reduction of the Error Due to DoF in AoA Measurement

Over a long period, publications concerning measurement of AoA have rarely considered controlling DoF as a source of error. A review in 1950 [44] proposed maximising test-object spatial frequency to reduce DoF in measuring AoA, and mentioned three previous proposals of a similar principle by different authors since 1885.

That principle does not appear to have been criticised in the literature. Nonetheless, only two reports [15,54] were found of the use of a method adopting that principle. No report was found of that principle being used in routine clinical work.

## 6. Conclusions

The error in AoA measurement due to DoF cannot be easily estimated or confidently predicted and can be large. Ideally, a new method of measurement should eliminate DoF and be suitable for routine clinical use.

## Figures and Tables

**Table 1:** AoA measurement: Studies showing the effect of reducing DoF.

Study Ref	How the Study Reduced the DoF	*n*	Ratio of Results with Reduced DoF to Results with a Standard Push-Up Method without Reduced DoF, for a Range of Age Groups				
			0–17	18–31	32–40	41–50	Over 51
[15]	Reduced test-object subtense	60		0.62	0.66	0.69	0.78
[26]	Stigmatoscopy	106				0.24	0.10
[38]	Minus lens method	61		0.76			
[39]	Minus lens method	125	0.74				
[39]	Retinoscopy	125	0.87				
[40]	Minus lens method	31			0.60	0.40	0.36
